# Dia- and Rok-dependent enrichment of capping proteins in a cortical region

**DOI:** 10.1242/jcs.258973

**Published:** 2021-11-05

**Authors:** Anja Schmidt, Long Li, Zhiyi Lv, Shuling Yan, Jörg Großhans

**Affiliations:** 1 Department of Biology, University of North Carolina at Chapel Hill, Chapel Hill, NC 27599, USA; 2 Department of Biology/FB17, Philipps University, Karl-von-Frisch-Straße 8, 35043 Marburg, Germany

**Keywords:** Cell cortex, Epithelial domain, Subapical, Formin, Dia, Actin, Capping protein, Myosin, *Drosophila*, Embryo

## Abstract

Rho signaling with its major targets the formin Dia, Rho kinase (Rok) and non-muscle myosin II (MyoII, encoded by *zip* in flies) control turnover, amount and contractility of actomyosin. Much less investigated has been a potential function for the distribution of F-actin plus and minus ends. In syncytial *Drosophila* embryos, Rho1 signaling is high between actin caps, i.e. the cortical intercap region. Capping protein binds to free plus ends of F-actin to prevent elongation of the filament. Capping protein has served as a marker to visualize the distribution of F-actin plus ends in cells and *in vitro*. In the present study, we probed the distribution of plus ends with capping protein in syncytial *Drosophila* embryos. We found that capping proteins are specifically enriched in the intercap region similar to Dia and MyoII but distinct from overall F-actin. The intercap enrichment of Capping protein was impaired in *dia* mutants and embryos, in which Rok and MyoII activation was inhibited. Our observations reveal that Dia and Rok-MyoII control Capping protein enrichment and support a model that Dia and Rok-MyoII control the organization of cortical actin cytoskeleton downstream of Rho1 signaling.

This article has an associated First Person interview with the first authors of the paper.

## Abstract (precis)

**Summary:** Plus ends of actin filaments are enriched at cortical regions rich in Rho signaling in syncytial *Drosophila* embryos depending on the actin regulator Dia and Rho kinase.

## INTRODUCTION

Underlying the plasma membrane of cells, a thin layer of cortical F-actin fulfills manifold functions such as playing a central role in cell polarity, providing a scaffold for membrane associated proteins, and ensuring mechanical stiffness, among others. Cortical F-actin contains F-actin regulators, Myosin motor proteins and other cortical proteins. Linker proteins belonging to the ERM protein family mediate attachment of cortical F-actin to the plasma membrane. The F-actin cortex contributes to segregation of proteins and establishment of cortical domains, including polarity proteins that are crucial for formation and maintenance of cell polarity (Honigmann and Pralle, 2016; Schmidt and Grosshans, 2018).

Cortical organization is dynamic during *Drosophila* development. Following a uniform cortex in preblastoderm stages, the cortex becomes patterned with the arrival of the nuclei at the cortex (blastoderm stages). During interphases, two cortical domains mark the cap region above the nuclei and intercap region in between caps (Karr and Alberts, 1986; Warn et al., 1984). During mitosis, metaphase furrows provide a physical barrier for the individual mitotic spindles. Three distinct cortical domains are observed during this process, namely apical, lateral and basal regions (Mavrakis et al., 2009).

Actin filaments contain an intrinsic polarity with their plus and minus ends or barbed and pointed ends, respectively. The polarity of filaments is reflected by numerous plus end or minus end binding proteins. The formin Diaphanous (Dia) specifically binds as a dimer to plus ends, where it catalyzes the incorporation of G-actin to promote filament elongation (Bogdan et al., 2013; Evangelista et al., 1997; Yan et al., 2013). Similarly Capping protein α (Cpa) tightly binds as a heterodimer with Capping protein β (Cpb) to plus ends of actin filaments (Amândio et al., 2014; Wear et al., 2003). In addition to plus end binding, the myosin motor protein non-muscle myosin II (MyoII, encoded by *zip* in flies) slides towards the plus ends of actin filaments (Houdusse and Sweeney, 2016) and induces formation of filament asters with plus ends at the center *in vitro* (Köster et al., 2016; Murrell and Gardel, 2012). The Tropomyosin-binding protein Tropomodulin (Tmod) binds to the minus ends of F-actin and has previously been employed as a marker for minus ends (Coravos and Martin, 2016; Fowler et al., 1993; Weber et al., 1994).

Dia and MyoII (via Rho kinase, Rok) are both controlled by Rho signaling. In early embryos, Dia is involved in the formation of pole cells, the metaphase furrow and the cellularization furrow, and together with Cip4 in the exclusion of lateral markers from the furrow canal (Afshar et al., 2000; Castrillon and Wasserman, 1994; Großhans et al., 2005; Yan et al., 2013).

Unlike actomyosin in muscle cells, the F-actin cortex is generally assumed to consist of a random meshwork of filaments with uniformly distributed plus ends and plus end binding proteins. In contrast, it is also expected that spatially patterned Rho1 signaling will impinge on the structure, dynamics and organization of the F-actin cortex. These contrasting views have not been much tested in a physiological context. In the present study, we compare the spatial pattern of Rho1 signaling in early *Drosophila* embryos with the distribution of the heterodimeric capping protein and test the function of two targets of Rho signaling, Dia and MyoII, in distribution of capping proteins.

## RESULTS

### Rho signaling is locally restricted during syncytial blastoderm development

**Fig. 1.**
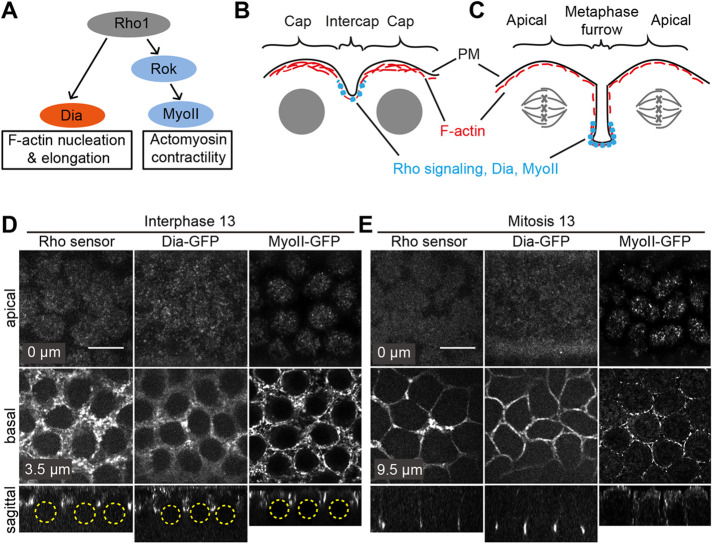
**Spatiotemporal pattern of Rho signaling.** (A) Scheme indicating the signal pathway downstream of Rho1, activating Dia that leads to F-actin nucleation and elongation and MyoII activation via ROCK that leads to actomyosin contractility. (B) Scheme of syncytial blastoderm interphase with F-actin-rich caps above nuclei and MyoII-rich intercap regions as indicated. (C) Scheme of syncytial blastoderm mitosis with metaphase furrows invaginating towards the interior of the embryo. Rho signaling, Dia and MyoII are enriched at the basal tip of the metaphase furrows. (D,E) Living wild-type embryos during syncytial blastoderm interphase (D) and mitosis (E) expressing a Rho sensor (Rho binding domain of anillin) tagged with GFP, Dia-GFP, or MyoII labeled with 3× GFP. Embryos were imaged from the top and apical-basal positions are as indicated within images. Computed sagittal views are shown in the bottom panels. Yellow dashed circles indicate the position of interphase nuclei. Scale bars: 10 µm.

Rho signaling controls F-actin in two ways, at least – nucleation and elongation via Dia and contractility via Rok and MyoII (Fig. 1A). The cortex of syncytial blastoderm *Drosophila* embryos is structured into two regions during interphase: actin caps and the region between actin caps, namely the intercap region (Fig. 1B). Both regions are differentially labeled by cortical markers (Schmidt et al., 2018). For example, MyoII is strongly enriched in the intercap region, whereas Arp2/3-dependent branched F-actin is highly enriched in the cap region (Stevenson et al., 2002). Using a genetically encoded Rho1 sensor (Munjal et al., 2015), we analyzed the spatiotemporal pattern of Rho signaling in syncytial blastoderm embryos. During interphase, we detected a strongly enriched Rho1 sensor signal in intercaps, but little in the cap region (Fig. 1D; Fig. S1). This finding is consistent with the intercap enrichment of MyoII as previously reported (Royou et al., 2002) and of Dia, assayed by GFP tagging (Fig. 1D; Figs S2 and S3). We labeled Dia by inserting a GFP tag before the stop codon by CRISPR-mediated integration at the endogenous *dia* locus (Fig. S2A,B). The Dia-GFP construct is fully functional, since we employed a homozygous stock, in which Dia-GFP completely substitutes Dia. It is worth noting that the pattern is rather uniform after Dia immunostaining in fixed embryos (Fig. S2C). The staining pattern of Dia-GFP depends on formin activity, since the intercap restriction was strongly reduced after treatment with the formin inhibitor SMIFH2, whereas overall F-actin caps were not affected by drug treatment (Fig. S4A,B).

As reported previously (Royou et al., 2002), we detected enriched Rho1 sensor and Dia-GFP signal at the tips of metaphase furrows during mitosis (Fig. 1C,E; Fig. S3) (Großhans et al., 2005; Padash Barmchi et al., 2005; Yan et al., 2013). Furthermore, we detected MyoII at the retracting metaphase furrow (Fig. 1E, right panel) (He et al., 2016). In summary, we revealed that Rho signaling and two targets are restricted to the intercap region during interphase. However, we did not test whether these proteins colocalize within the region of the basal tip.

### Cpa is enriched at the intercap region in a *dia*-dependent manner

Having identified a restriction of Rho signaling and two of its targets to the intercap region, we analyzed markers for the organization of cortical F-actin. We found that the plus-end binding proteins Cpa and Cpb as well as the minus-end binding protein Tmod were enriched at intercaps.

**Fig. 2.**
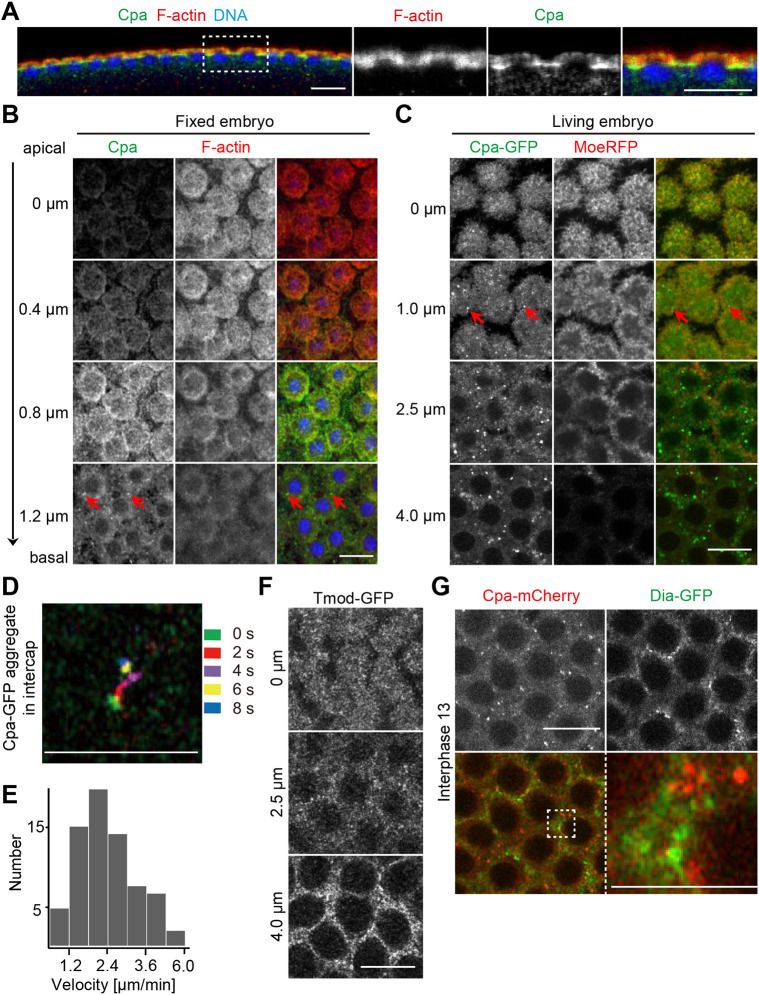
**Cpa and CpaGFP are enriched at intercap regions.** (A,B) Embryos in interphase 13 fixed and stained for Cpa (green), F-actin (red) and DNA (blue). The region marked with a dotted rectangle is shown in high magnification on the right. (A) Sagittal section. (B) Frontal sections. Pictured are top views arranged along the apical-basal axis with positions as indicated. Red arrows indicate Cpa puncta. (C) Live embryo expressing CpaGFP (green) and Moesin-RFP (MoeRFP, red). Interphase 13. Frontal sections along apical-basal axis are shown as indicated. Red arrows indicate CpaGFP puncta. (D) Track of a single cortical cap CpaGFP puncta represented by colors over time as indicated. (E) Dynamics of CpaGFP puncta. (F) Live embryo expressing Tmod-GFP to mark F-actin minus ends. Displayed are stills along the apical-basal axis as indicated. (G) Images from live embryos expressing Cpa-mCherry (red) and Dia-GFP (green) during interphase 13. The region marked with a dotted square is shown in high magnification on the right. Scale bars: 10 µm. Scale bars in D and right-hand panel of G: 5 µm. Results are representative of more than three experiments.

Cpa immunostaining revealed a strong enrichment outside of actin caps in intercaps during syncytial blastoderm interphases distinct from overall F-actin distribution (Fig. 2A). A similar distribution was detected by immunostaining against Cpb (Fig. S4C). The distinct distribution pattern of Cpa and overall F-actin became also obvious in axial sectioning of frontal views (Fig. 2B). Staining of actin caps was prominent in the upper layers, whereas Cpa staining was more prominent in lower sections beside the caps, indicating an enrichment within the intercap regions. Besides a diffuse staining, we detected prominent puncta of a wide range of intensities, especially at basal positions, which may represent Cpa clusters (Fig. 2B, red arrows). The prominence and appearance of the puncta depended on fixation conditions (Fig. S4D–F).

We confirmed the intercap enrichment by live imaging with a C-terminally tagged Cpa at the endogenous locus (Fig. 2C; Figs S4G and S5). In frontal views, we observed an enrichment of the CpaGFP puncta in regions of basal sections outside of actin caps beyond a diffuse staining of caps and intercaps overlapping with the F-actin label Moesin-GFP. The puncta appeared more prominent than in fixed embryos (Fig. 2C, red arrows). The tagged Cpa proteins were functional. Homozygous stocks containing only Cpa molecules at comparable expression levels were viable and fertile (Fig. S4H,I). The GFP or Cherry tagging did not affect subcellular distribution, since the staining pattern of fixed wild-type and CpaGFP embryos was comparable (Fig. S4C). The Cpa puncta were mobile, with a distribution of their velocities in the range of a few µm/min (Fig. 2D,E), which is in the range of cytoskeleton-dependent movements (Theriot and Mitchison, 1991).

We also detected an intercap enrichment of the minus end marker Tmod during interphase on top of an overall cortical labeling (Fig. 2F; Fig. S6). We assayed Tmod distribution in living embryos with a functional GFP fusion protein, expressed from the endogenous locus (Coravos and Martin, 2016). Consistent with the typical length of F-actin, we detected both plus and minus end markers within the intercap region. In the following experiments, we focused on the plus end marker Cpa.

Given that both Dia and Cpa bind to plus ends of actin filaments and both are enriched in intercaps, we directly compared their distribution in living embryos coexpressing Dia-GFP and Cpa-mCherry (Fig. 2G). We did not detect an obvious overlap of the prominent Cpa-mCherry and Dia-GFP puncta, suggesting that Cpa and Dia interact with distinct populations of F-actin. However, we do not exclude some degree of colocalization given the weak uniform cortical labeling of both proteins.

**Fig. 3.**
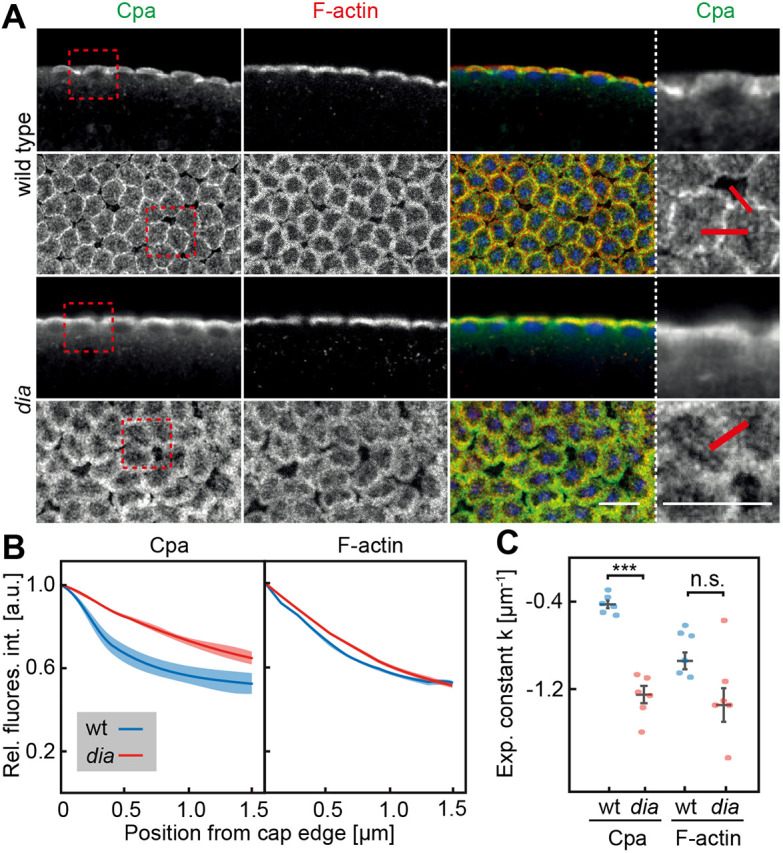
**Cpa enrichment depends on *dia*.** (A) Wild-type and dia embryos in interphase 13 fixed and stained against Cpa (green), F-actin (red) and DNA (blue). Sagittal sections are shown in the top panels, frontal sections in the bottom panels. Regions marked by dotted squares are magnified in panels on the right. Red lines indicate the measurement by line profiles. Scale bars: 10 µm. (B) Averaged and normalized intensity profiles of wild-type (wt, blue) and *dia* (red) interphase 13 embryos across cap edge, as indicated in (A). Fluorescence intensities of three embryos per genotype were measured (wild type 98 edges, *dia* 108 edges in total) across cap-intercap borders normalized to their peak and mirrored at the peak. s.e.m. is indicated by transparent regions. (C) Fitted exponential constants of intensity profiles per embryo for Cpa and F-actin signal were plotted. Mean values and s.e.m. are shown by bars. ***P≤0.001 (*P*-value was determined by two-tailed *t*-test).

Next, we tested whether intercap enrichment of Cpa depended on *dia* in fixed and live embryos (Fig. 3). We generated *dia* mutant embryos from *dia^SY5^* germline clones, which is a missense mutation within the N-terminal Rho binding domain (Yan et al., 2013), and contains very little Dia protein (Fig. S2C,D). Strikingly, Cpa was almost uniformly distributed at the cortex in *dia* mutants, including actin caps as compared to the sharp lines representing the intercap regions (Fig. 3A). In order to compare wild-type and mutant staining patterns in quantitative terms, we measured the distribution of the Cpa signal by line profiles in comparison to F-actin (Fig. 3B). We quantified fluorescence intensities across cap-intercap and cap-cap edges and normalized and aligned the curves to their maxima with 0 µm representing the cap-cap or cap-intercap border. We mirrored the data at the maxima to obtain a curve with decreasing fluorescence intensity to which we fitted an exponential function. The resulting exponential decay constant κ describes the decrease of fluorescence intensity over the distance away from the maxima either towards the cap center or towards the cap outside. The average exponential decay constant in wild-type caps was significantly higher than in *dia* caps (Fig. 3C), which indicates a broader distribution in *dia* mutants. The F-actin signal has the maximum also at the edge between caps and intercap regions because of axial distortion of the focal volume and curved morphology of the caps in interphase 13. As clearly visible by the loss of the sharp lines of Cpa staining in *dia* mutants, we measured a significant difference for the Cpa distribution between wild type and *dia* mutants, whereas F-actin distribution was statistically not significantly different (Fig. 3B,C).

**Fig. 4.**
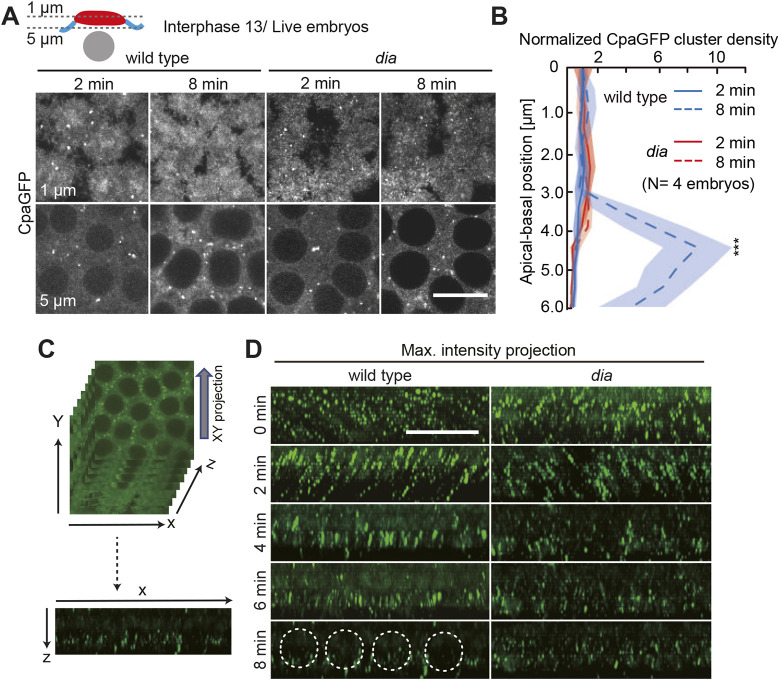
**Basal accumulation of CpaGFP puncta depends on *dia*.** (A) Live wild-type and *dia* embryos during interphase 13 expressing CpaGFP. The top panels display a z-section of the actin caps (1 µm), whereas the bottom panels show a z-section of the intercap region (5 µm), as indicated in the schematic representation, during early (2 min) and late (8 min) interphase. (B) Apical (cap) to basal (intercap) distribution of CpaGFP clusters within the field of view of four imaged embryos per genotype as indicated. The number of clusters was normalized to the first apical slice. Solid lines represent early interphase (2 min), dashed lines late interphase (8 min) in wild-type (blue) and *dia* (red) embryos. (C) To obtain maximum intensity projections shown in D, z-stacks of frontal sections were projected as orthogonal views. (D) Orthogonal views of wild-type (left) and *dia* (right) embryos expressing CpaGFP during interphase 13 at indicated time points. The positions of nuclei are indicated by dashed circles. Scale bars: 10 µm.

We also confirmed the dependence of the accumulation of basal CpaGFP puncta on *dia* by live imaging during interphase 13. In wild-type embryos, we could detect a basal accumulation over time in axial slices covering the intercap region in contrast to *dia* embryos (Fig. 4A). We quantified the basal accumulation of CpaGFP puncta along the apical-basal axis over time by counting the number of puncta in the field of view in four embryos per genotype and normalized to the most apical region to compare the density of puncta. The normalized density was elevated in wild-type intercap regions compared to *dia* intercaps (Fig. 4B). To evaluate if the CpaGFP puncta were overall diminished in dia embryos, we computed orthogonal views from axial stacks resulting in maximum intensity projections displaying the apical-basal distribution of CpaGFP (Fig. 4C). In wild-type embryos we could detect a basal accumulation of the CpaGFP puncta over time, whereas the CpaGFP puncta in *dia* embryos remained distributed along the whole apical-basal axis (Fig. 4D).

### Cpa enrichment depends on Rok activity

**Fig. 5.**
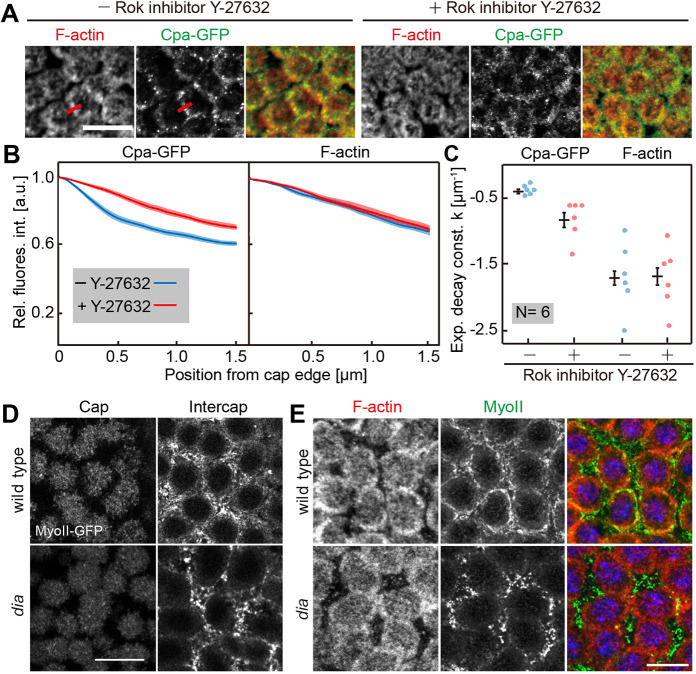
**MyoII controls Cpa distribution.** (A) Fixed embryos in interphase 13 injected with or without Rok inhibitor Y-27632, fixed and stained for F-actin (red) and CpaGFP (green). Red lines indicate the measurement by line profiles shown in B. (B) Normalized fluorescence intensity profiles of CpaGFP and F-actin in Y-27632-injected and control embryos. Six embryos were quantified per genotype (56 edges in control, 63 edges in Y-27632-injected embryos in total). Average is shown as a solid line, s.d. as a band. (C) Fitted exponential constant from intensity profiles of injected and control embryos. Corresponding averages and s.d. are shown by bars. **P≤0.001 (*P*-value was determined by two-tailed *t*-test). (D) Live wild-type and *dia* embryos expressing MyoII-GFP in interphase 13. Frontal sections of the cap and intercap layers are shown. (E) Wild-type and *dia* embryos in interphase 13 fixed and stained for F-actin (red) and MyoII-GFP (green). The images are maximum intensity projections. Scale bars: 10 µm.

As Rho signaling controls both Dia and MyoII in parallel, we hypothesized that MyoII may also control Cpa enrichment at intercaps. We indirectly inhibited MyoII by injecting the Rho kinase inhibitor Y-27632 (Fig. S4A) (Ishizaki et al., 2000). We cannot exclude that other targets of Rok besides MyoII contribute to the observed phenotypes. We detected a loss of Cpa enrichment after inhibitor injection (Fig. 5A), reminiscent to the phenotype in *dia* mutants. Quantification of line profiles revealed a significant difference for Cpa but not F-actin after Rok inhibition (Fig. 5B,C). Dia and MyoII function in parallel according to the standard model of Rho signaling (Piekny et al., 2005). We tested for a mutual dependence by analyzing MyoII distribution in *dia* mutants and Dia-GFP in Rok-inhibited embryos (Fig. 5; Fig. S4A). The overall MyoII levels and restriction to the intercap region appeared comparable between wild type and *dia* mutants. Yet, the staining in the submicron scale appeared grainier, which could be caused by a partially disrupted and less continuous actomyosin network in the intercap regions (Fig. 5D,E) (Jiang and Harris, 2019). We did not observe a change in the pattern of Dia-GFP following Rok inhibition (Fig. S4A,B). We conclude that Cpa depends on both targets of Rho signaling: Rok-MyoII activity and Dia.

### Cpa distribution in embryos lacking caps

**Fig. 6.**
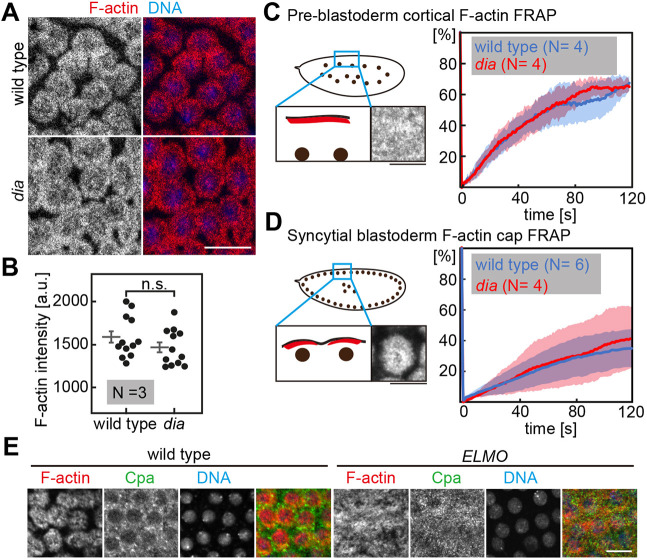
**F-actin amount and dynamics in *dia* mutants.** (A) Wild-type and *dia* embryos fixed and stained against F-actin (red) and DNA (blue). (B) Quantification of F-actin signal in A as indicated. In total, 12 frames of three embryos per genotype were quantified. Corresponding averages and s.e.m. are shown by bars. *P* not significant (n.s.) as determined by two-tailed *t*-test. (C,D) FRAP in pre-blastoderm (C) and syncytial blastoderm (D) embryos expressing utrophin-GFP. Schematic representations of embryonic stages in frontal sections of utrophin-GFP are shown on the left. Graphs represent the averaged percentage of fluorescence recovery over time in wild-type (blue) and *dia* (red) embryos. For C, four embryos per genotype were tested; for D, 6 wild-type and 4 *dia* embryos were tested. s.d. are represented by transparent regions along the curves. Scale bars: 10 µm. (E) Wild-type and *ELMO* germline clone embryos in interphase 13 fixed and stained for F-actin (red), Cpa (green), and DNA using DAPI (blue). Scale bars: 10 µm.

The intercap enrichment of Cpa is likely an indirect consequence of the function of Dia and Rok-MyoII on actomyosin amount, turnover, organization or activity. The changed Cpa enrichment in *dia* mutants may be a consequence of altered F-actin dynamics and amounts. To address this option, we quantified cortical F-actin in wild type and *dia* mutants by measuring overall fluorescence intensity of 4 frames per embryo in three embryos per genotype. Wild-type and *dia* embryos were stained and imaged within the same batch and distinguished by the expression of MyoII-GFP by *dia* embryos. The frames were imaged in an axial position that captured the actin caps. We did not detect a difference in staining with phalloidin (Fig. 6A,B). *dia* mutants have rudimentary metaphase furrows and have been reported to have slightly smaller caps (Cao et al., 2010).

We also did not detect changes in the turnover of F-actin in *dia* embryos. F-actin turnover was measured by fluorescence recovery after photobleaching (FRAP) with Utrophin-GFP, which stably binds to F-actin allowing assay of the exchange kinetics (Burkel et al., 2007) and serving as a good proxy for F-actin dynamics by FRAP. In both the uniform cortex of pre-blastoderm embryos and the actin caps of blastoderm embryos (Fig. 6C,D), we did not detect any obvious difference between wild-type and *dia* embryos. However, we could detect a higher variability in syncytial blastoderm *dia* mutants compared to the fluorescence recovery in wild-type embryos. Taken together, our data indicate that total F-actin amount and dynamics at the cortex is not obviously changed in preblastoderm and syncytial blastoderm *dia* mutants.

The cortex of syncytial embryos is set by actin caps, which are controlled by Rac signaling and Arp2/3-dependent F-actin (Stevenson et al., 2002). We tested how the pattern of Cpa depended on the actin caps by analyzing embryos lacking actin caps. Rac signaling in the cap region is controlled by a complex of ELMO (also known as Ced-12) and Sponge, which constitute an unconventional guanyl nucleotide exchange factor (Postner et al., 1992; Stevenson et al., 2002; Zallen et al., 2002; Zhang et al., 2018). Embryos from *ELMO* germline clones, similar to embryos from *sponge* females, display a largely uniform actin cortex without actin caps (Postner et al., 1992; Schmidt et al., 2018; Winkler et al., 2015). In fixed embryos from *ELMO* germline clones, we detected a largely uniform distribution of Cpa staining comparable to the distribution of F-actin (Fig. 6E). We could detect Cpa puncta in *ELMO* mutants, although they seemed smaller in size and did not display a specific localization compared to the clear intercap localization in wild type. We conclude that Cpa is enriched at intercaps by an exclusion from the cap region.

## DISCUSSION

We have revealed that Cpa distribution is surprisingly uneven and divergent from overall F-actin in syncytial blastoderm embryos. Cpa is enriched in areas of Rho1 signaling in a manner depending on the Rho1 targets Dia and Rok. Although cortical organization in syncytial embryos is special, we speculate that Cpa is also differentially distributed within the cell cortex of other cell types as well as in species other than *Drosophila*. All components, i.e. Rho GTPase, Dia, Rok, MyoII and the Cpa–Cpb complex, have been highly conserved through evolution.

### Cpa is enriched in regions with Rho signaling

On top of an expected uniform and diffuse staining largely matching global F-actin distribution, we detected an enrichment of Cpa at the intercaps. A corresponding restriction to the intercap region is observed for Rho1 signaling and two of its targets, Dia and Rok-MyoII. Besides levels, turnover and contractility of actomyosin, we wondered whether organization of cortical actin would depend on Dia and Rok-MyoII. To assay the organization of cortical actin we employed the plus end binding proteins Cpa and Cpb. Cpa has been used previously to assay the organization of F-actin in cells of the mesoderm anlage (e.g. Coravos and Martin, 2016). We detected an enrichment of Cpa staining in the intercap region of fixed and live embryos, despite the low total F-actin levels compared to the actin caps. Cpa staining is visible in prominent puncta beyond a diffuse staining. The puncta are more obvious in live than fixed embryos, which may be a result of sensitivity to fixation conditions of the underlying structure. Given their dynamics and mobility, the puncta may represent sites of interaction between astral microtubules and the actin cortex. Other speculative options for the puncta are the ends of actin bundles or centers of asters. Further experiments, especially involving nanoscopic analysis, may provide insight into the nature and interaction of the Cpa clusters.

Taking a simple-minded view with Cpa and Tmod as markers of filament ends, the observed enrichment can be interpreted as a preferential accumulation of F-actin plus and minus ends in the intercap region. Our staining experiments and measurements indicate that the ratio of filament ends versus total amount of F-actin is strongly increased in the intercap region. However, the interpretation and underlying causes may be more differentiated and complicated. One explanation is that filament ends are not equally accessible to the Cpa–Cpb complex and Tmod. For example, the cap region largely contains Arp2/3-dependent branched F-actin, which by structure contain fewer minus ends. The plus ends within the caps may have a reduced preference for Cpa binding or may be subject to competition from other, branched actin specific, regulators. Alternatively, the filaments may be much shorter in the intercap than cap region. *In vitro* studies showed that myosin-based contractility may lead to severing of actin filaments (Murrell and Gardel, 2012). A consequence of the Rho1 signaling and MyoII activation in the intercap region may be increased contractility and filament severing. Shorter filaments may also arise from dedicated severing proteins like cofilin or from increased actin turnover. Despite these complications, the observed enrichment of markers for filament ends indicates a specific feature of cortical actin organization at the intercap region involving plus ends.

### Dia and MyoII regulate cortical actin organization

Having identified an intercap enrichment, we characterized the underlying mechanism. We revealed a novel common function of Dia and MyoII for the enrichment of capping proteins in intercaps. Surprisingly, we found that both targets of Rho signaling, Dia and Rok-MyoII, are required in an apparently parallel fashion.

With both proteins binding to plus ends, Cpa and Dia are expected to show an interesting interaction. While some previous reports indicate a mutual exclusion at the plus ends (Bartolini et al., 2012; Kovar et al., 2005), other reports suggest them to be cross regulators that can both simultaneously bind to the plus end and accelerate dissociation of one of the proteins (Shekhar et al., 2015). Concerning the function, it is conceivable that, on the one hand, Dia acts directly on binding and thus orienting plus ends, without a contribution by Cpa. On the other hand, Cpa and Dia may directly cooperate in orienting actin filaments. We did not detect an overlap of Cpa-mCherry clusters and Dia-GFP in our time-lapse recordings, whereas the weaker diffuse Cpa signal is overlapping with Dia-GFP clusters.

Similar to the relation of Dia to capping proteins, we discovered a corresponding function of Rok and possibly also of its major target MyoII. A role of MyoII in organization of actin filaments *in vitro* is well established. Reconstituted F-actin systems lead to asters with MyoII and F-actin plus ends in their center, as demonstrated with Cpa as a plus-end label (Köster et al., 2016; Wollrab et al., 2019). Such an orientation is consistent with the movement of MyoII molecules towards the plus ends of the actin filaments by acting as a crosslinker and sliding actin filaments until reaching their plus ends (Houdusse and Sweeney, 2016). By revealing a common function for both MyoII and Dia in enrichment of capping protein, the question of their functional relationship arises. We did not detect a mutual dependence of Dia and Rok-MyoII localization. Although we observed a slightly changed pattern, MyoII clusters were still present in *dia* mutants, suggesting that Dia and MyoII act in parallel in a non-redundant manner. The parallel control and the biochemical activities of Dia and MyoII suggest that they do not directly recruit Cpa to the intercap region but likely act indirectly via the structure of F-actin.

In accordance with *in vitro* studies as mentioned above (Köster et al., 2016; Wollrab et al., 2019), we propose a speculative model where Dia molecules, activated by Rho signaling, serve as fixpoints for a fraction of actin filaments and orient the filaments within the cortex. In parallel, MyoII motor proteins are activated by Rho signaling via Rok within the same regions. Thus, both branches of Rho signaling lead to an accumulation of plus ends within the signaling region. A corresponding model concerning Dia has been proposed for the apical cortical F-actin during apical constriction during mesoderm invagination, where Dia contributes to orientation of cortical F-actin towards the adherens junctions (Coravos and Martin, 2016).

### What might be the function of enriched capping protein?

A function for the intercap enrichment of Cpa is not immediately obvious and we can only speculate about conceivable functions. The most obvious morphological function of Dia and to a lesser degree MyoII is in the formation and elongation of the metaphase furrow during nuclear divisions. *dia* mutants display short metaphase furrows resulting in perturbed spindle positioning and multinuclear cells (Afshar et al., 2000). A corresponding function of Rok-MyoII activity is less pronounced, possibly because of compensation by Anillin and Septin (Royou et al., 2004; Zhang et al., 2018). It is conceivable that the growth of the metaphase furrow is mediated by oriented actin filaments leading to growth and buckling of the caps (Zhang et al., 2018).

During interphase, Dia is involved in the dynamics and lateral repositioning of the nuclei after mitosis (Kanesaki et al., 2011; Lv et al., 2020). The yoyo movement of nuclei is impaired in *dia* mutants, in that the return movement is reduced or missing. In general, the actin cortex is assumed to provide mechanical stability to the cell membrane. The viscoelastic properties of the embryonic cortex and membrane have not been assessed concerning a function of Dia or MyoII. Suitable biophysical measurements will be required to test for such a function.

Lastly, the enrichment of capping proteins may be involved in segregation of cortical markers, although we have not yet detected an impaired segregation of markers for caps and intercaps. Despite this, it is possible to speculate that the orientation of actin filaments is used as a template for the more elaborate cortical pattern in the following developmental step, i.e. cellularization.

Taken together, we revealed a novel function of Dia and Rok-MyoII in enrichment of capping proteins and of F-actin plus ends if interpreting Cpa as a plus end probe. Further studies will be necessary to determine if an uneven, anisotropic distribution of capping proteins and F-actin plus ends could be a generic feature of the cell cortex found also in other developmental stages and systems.

## MATERIALS AND METHODS

### Fly stocks and handling

Fly stocks used were *Ced-12/ELMO^c06760^* (Bloomington *Drosophila* Stock Center), CpaGFP, Cpa-mCherry and Dia-GFP (the present study), *dia^sy5^* (Yan et al., 2013), Histone2Av-GFP (Clarkson and Saint, 1999), Moesin-RFP (Großhans lab, *w*; P{w^+^, sqh-moesin-RFP}), MyoII3xGFP (Pinheiro et al., 2017), Rho1 sensor (Ubi-Anillin-RBD-EGFP) (Munjal et al., 2015), Tmod-GFP (Bloomington *Drosophila* Stock Center), and utrophin-GFP (Rauzi et al., 2010) (Table S1).

Histone2Av-GFP was used for scoring wild-type embryos in staining experiments with mixed genotypes. *w^1118^* was used as a wild-type control. All fly stocks were obtained from the Bloomington *Drosophila* Stock Center (Whitworth, 2019), unless stated otherwise. Genetic markers, annotations and their references are described in Flybase.org (Gramates et al., 2017).

All crosses and cages were kept at 25°C. Germline clones were produced and selected by the ovo-flippase technique, as described previously (Chou et al., 1993).

Targeted insertions, CpaGFP, Cpa-Cherry and Dia-GFP, were generated by CRISPR/Cas9 (InDroso Functional Genomics, Rennes, France; WellGenetics, New Taipei City, Taiwan). The coding sequence of eGFP or mCherry with a 5′-terminal linker sequence was inserted into the 3′ region of the target gene.

The gene region of CpaGFP is as follows (EXON, intron, **LINKER**, *egfp; UTR*): 5′-CCAGTTGCCCATCACCAGGACCAAGATCGACTGGAGCAAAATCGTCTCGTACAGCATTGGCAAGGAACTGAAGACGCAA**GGCGTGGGC***atggtgagcaagggcgaggagctgttcaccggggtggtgcccatcctggtcgagctggacggcgacgtaaacggccacaagttcagcgtgtccggcgagggcgagggcgatgccacctacggcaagctgaccctgaagttcatctgcaccaccggcaagctgcccgtgccctggcccaccctcgtgaccaccctgacctacggcgtgcagtgcttcagccgctaccccgaccacatgaagcagcacgacttcttcaagtccgccatgcccgaaggctacgtccaggagcgcaccatcttcttcaaggacgacggcaactacaagacccgcgccgaggtgaagttcgagggcgacaccctggtgaaccgcatcgagctgaagggcatcgacttcaaggaggacggcaacatcctggggcacaagctggagtacaactacaacagccacaacgtctatatcatggccgacaagcagaagaacggcatcaaggtgaacttcaagatccgccacaacatcgaggacggcagcgtgcagctcgccgaccactaccagcagaacacccccatcggcgacggccccgtgctgctgcccgacaaccactacctgagcacccagtccgccctgagcaaagaccccaacgagaagcgcgatcacatggtcctgctggagttcgtgaccgccgccgggatcactctcggcatggacgagctgtataagTAAGACCAGGAGGTGCAAGCGGAAGAGGAGCGGACACAGTCGACAAAGCGGCCCAATAGTCTTCCACTTGCCATGGCCAAGCGAGCATAGGAACCGATCAC*-3′.

Cpa-mCherry (EXON, **LINKER**, *mcherry, LoxP*): 5′-CGACATTCAAGGCAATGCGTCGCCAGTTGCCCATCACCAGGACCAAGATCGACTGGAGCAAAATCGTCTCGTACAGCATTGGCAAAGAACTGAAGACGCAA**TCTAGA***atggtgagcaagggcgaggaggataacatggccatcatcaaggagttcatgcgcttcaaggtgcacatggagggctccgtgaacggccacgagttcgagatcgagggcgagggcgagggccgcccctacgagggcacccagaccgccaagctgaaggtgaccaagggtggccccctgcccttcgcctgggacatcctgtcccctcagttcatgtacggctccaaggcctacgtgaagcaccccgccgacatccccgactacttgaagctgtccttccccgagggcttcaagtgggagcgcgtgatgaacttcgaggacggcggcgtggtgaccgtgacccaggactcctccctgcaggacggcgagttcatctacaaggtgaagctgcgcggcaccaacttcccctccgacggccccgtaatgcagaagaagaccatgggctgggaggcctcctccgagcggatgtaccccgaggacggcgccctgaagggcgagatcaagcagaggctgaagctgaaggacggcggccactacgacgctgaggtcaagaccacctacaaggccaagaagcccgtgcagctgcccggcgcctacaacgtcaacatcaagttggacatcacctcccacaacgaggactacaccatcgtggaacagtacgaacgcgccgagggccgccactccaccggcggcatggacgagctgtacaagtagCTGCAGATAACTTCGTATAATGTATGCTATACGAAGTTATGCTAGC*-3′.

Dia-GFP (EXON, **LINKER**, *egfp, LoxP*): 5′-CGGACGCGTGTCACCAACGGACAACTAATGACCCGCGAAATGATCCTCAACGAGGTTCTAGGCTCCGCG**TCTAGA***atggtgagcaagggcgaggagctgttcaccggggtggtgcccatcctggtcgagctggacggcgacgtaaacggccacaagttcagcgtgtccggcgagggcgagggcgatgccacctacggcaagctgaccctgaagttcatctgcaccaccggcaagctgcccgtgccctggcccaccctcgtgaccaccctgacctacggcgtgcagtgcttcagccgctaccccgaccacatgaagcagcacgacttcttcaagtccgccatgcccgaaggctacgtccaggagcgcaccatcttcttcaaggacgacggcaactacaagacccgcgccgaggtgaagttcgagggcgacaccctggtgaaccgcatcgagctgaagggcatcgacttcaaggaggacggcaacagcctggggcacaagctggagtacaactacaacagccacaacgtctatatcatggccgacaagcagaagaacggcatcaaggtgaacttcaagatccgccacaacatcgaggacggcagcgtgcagctcgccgaccactaccagcagaacacccccatcggcgacggccccgtgctgctgcccgacaaccactacctgagcacccagtccgccctgagcaaagaccccaacgagaagcgcgatcacatggtcctgctggagttcgtgaccgccgccgggatcactctcggcatggacgagctgtacaagtaaCTGCAGATAACTTCGTATAATGTATGCTATACGAAGTTATGCTAGC*-3′.

### Immunostaining and antibodies

Following primary antibodies were used: mouse anti-α-Tubulin (1:50,000 for western blotting, B512, Sigma); rabbit anti-Cpa (1:200, 1:2000 for western blotting; Amândio et al., 2014 and the present study); rabbit anti-Cpb (1:200, Amândio et al., 2014), rabbit anti-Dia (1:1000, Großhans et al., 2005), guinea pig anti-Dia (1:5000 for western blotting, Großhans et al., 2005). F-actin was stained using phalloidin coupled to Alexa Fluor 647 (Thermo Fisher Scientific). Secondary antibodies for immunostaining experiments were labeled using Alexa Fluor 488, 568 and 647 (Thermo Fisher Scientific). The staining of GFP was done by making use of GFP-booster coupled with Atto488 (1:500, Chromotek). DNA was stained using DAPI (0.2 µg/ml, Thermo Fisher Scientific). Secondary antibodies for western blots were IRDye-800CW and IRDye-680 (1:20,000, LI-COR Biotechnology).

The full length Cpa coding sequence was cloned into pGEX-His, leading to a GST domain at the N-terminus and a 6× His tag at the C-terminus. The Cpa antibody was raised in rabbit against recombinant GST-Cpa-His6 protein expressed in *Escherichia coli* and purified under denaturing conditions according to standard protocols (Qiagen).

For histological staining, embryos were fixed by formaldehyde or heat fixation using standard methods described previously (Großhans et al., 2005). After fixation, embryos were stored in methanol at −20°C. Embryos for manual devitellinization were fixed by 8% formaldehyde (F-actin or Cpa staining). Fixed embryos were transferred to PBT (PBS+0.2% Tween20), washed three times for 5 min and then blocked for 30–60 min in PBT+5% bovine serum albumin (BSA). Incubation with primary antibodies was performed in PBT+0.1% BSA overnight at 4°C or for 2–3 h at room temperature. Afterwards, the embryos were washed with PBT three times for 15 min before incubation with secondary antibodies in PBT for 1–2 h at room temperature followed by three washing steps with PBT for 15 min. DNA staining by DAPI was performed for 10 min at room temperature, followed by washing for 15 min. The embryos were mounted in Aqua-Poly/Mount (Thermo Fisher Scientific).

The ROCK inhibitor Y-27632 (Sigma) was injected into embryos expressing CpaGFP during preblastoderm at a concentration of 10 mM. Embryos were fixed after reaching syncytial blastoderm and stained as described above after hand-removal of the vitelline membrane.

Details on antibodies, stains and inhibitors are given in Table S2.

### Western blotting

Embryo extracts were produced by freezing dechorionated embryos in liquid nitrogen. The frozen embryos were homogenized with a pestle in 2× Laemmli buffer to generate an extract with 1.5 embryos/µl. The extract was heated to 95°C for 10 min and loaded onto a 10% SDS gel. Blotting onto a nitrocellulose membrane was performed for 1.5 h and the membrane was then blocked for 1 h in PBS+5% milk powder. Incubation with primary antibodies in PBT was performed overnight at 4°C followed by washing four times with PBT and incubation with the secondary antibodies for 2 h at room temperature in PBT. The membrane was washed four times with PBT and then imaged using an Odyssey CLx infrared imaging system (LI-COR Biosciences) with 16-bit depth. Images were processed using Adobe Photoshop and Illustrator.

### Imaging and quantifications

Imaging was performed using a Zeiss LSM 780 confocal microscope equipped with Airyscan or LSM980 Airyscan 2. Fixed samples were imaged using an LCI Plan Neofluar 63×/water NA 1.3 objective and live imaging was performed with a Plan Neofluar 63×/oil NA 1.4 objective. Embryos for live imaging were handled as described previously (Kanesaki et al., 2011). Embryos expressing the Rho biosensor were live imaged with a frame size of 512×512 pixels (33.7×33.7 µm; 70 nm lateral pixel size) and z-stacks were conducted with a step size of 0.5 µm. Orthogonal views were made with Fiji/ImageJ (Schindelin et al., 2012). Planar XY projections were generated with IMARIS from axial image stacks of CpaGFP (Fig. 4C). Wild-type or *dia* germline clone embryos expressing CpaGFP were live imaged with a frame size of 488×488 pixels (32.16×32.16 µm), a z-stack step size of 0.5 µm and a frame rate of 120 s.

F-actin in fixed wild-type and *dia* embryos (Fig. 6) was quantified by the fluorescence intensity of four frames with a size of 256×256 pixels per embryo. The embryos of both genotypes were stained in the same tube and imaged with the same settings. The genotypes were discriminated by a fluorescent tag, which was expressed by *dia* embryos. Three embryos per genotype were used for quantification.

FRAP experiments were conducted with wild-type and *dia* germline clone embryos expressing utrophin-GFP (Fig. 6C,D). The bleaching and recording were carried out with an Axio Observer microscope (Zeiss) equipped with a spinning disc (40×/NA1.3 oil) and an additional diode laser (473 nm, 100 mW; Rapp OptoElectronic, Hamburg, Germany). Bleaching of utrophin-GFP was carried out in a 5×5 µm region within a single actin cap using 60% laser power output for 1 s during the image acquisition. The images were taken for 2–3 min with a frame rate of 1 s/frame. To quantify the mobile fraction of F-actin, the fluorescence intensity in the bleaching region was measured in Fiji/ImageJ manually. The fluorescence intensity was normalized as follows: Normalized intensity: I=(I_t_−I_min_)/(I_max_−I_min_). Where I_t_ is the intensity at time t, I_min_ is the intensity immediately post-bleach and I_max_ is the intensity pre-bleach.

The formin inhibitor SMIFH2 (Abcam) was dissolved in DMSO with a concentration of 2 mM and injected into syncytial embryos expressing Dia-GFP (Fig. S4A). The fluorescence intensity of Dia-GFP in living embryos during interphase 13 with or without SMIFH2 treatment was measured with frames of 28.0×1.67 µm at the intercap region. The fluorescence intensities of Dia-GFP were normalized.

To track the movement of CpaGFP puncta (Fig. 2D), living embryos expressing CpaGFP during interphase on the cap region were recorded with a frame size of 488×488 pixel (21.44×21.44 µm), a z-stack step size of 0.25 µm and a frame rate of 2 s.

The dynamics of Cpa puncta in CpaGFP embryos during interphase (Fig. 2E) was quantified by measuring the distance that Cpa puncta move in 2 s on the cap region with a projection of 0.5 µm in Fiji/ImageJ manually. In total, the movements of 73 Cpa puncta were measured.

The fluorescence intensity of Cpa in fixed embryos (Fig. 3) was quantified by measuring fluorescence intensities across cap edges with a line width of 10 pixels. For both genotypes, three embryos in interphases 11–13 were used. The fluorescence intensities of each cap edge were normalized to their individual peak and all aligned to 1, which is position 0 µm in the plots. Averages of all measurements in each embryo were plotted on a graph. Each averaged quantification was mirrored at position 0 µm and subjected to exponential fitting (OriginPro 8.5G, GraphPad Prism 6) in a region from position 0–1.5 µm.

The density of CpaGFP puncta (Fig. 4B) was determined by quantification of puncta in each z-slice along the apical-basal axis. The numbers of puncta were normalized to 1 in the first slice.

Embryos expressing Dia-GFP or MyoII-GFP (Fig. S4) were collected and injected with the ROCK inhibitor Y-27632. Imaging was performed using a spinning disk microscope 20 min after injection.

## Supplementary Material

Supplementary information

Reviewer comments
