# Supplementary material for: Dia- and Rok-dependent enrichment of capping proteins in a cortical region
Source: J Cell Sci. 2021 Nov 5;134(21):jcs258973. doi: 10.1242/jcs.258973 (PMC8627554; doi:10.1242/jcs.258973)
Supplement: Supplementary information [file joces-134-258973-s1.pdf]

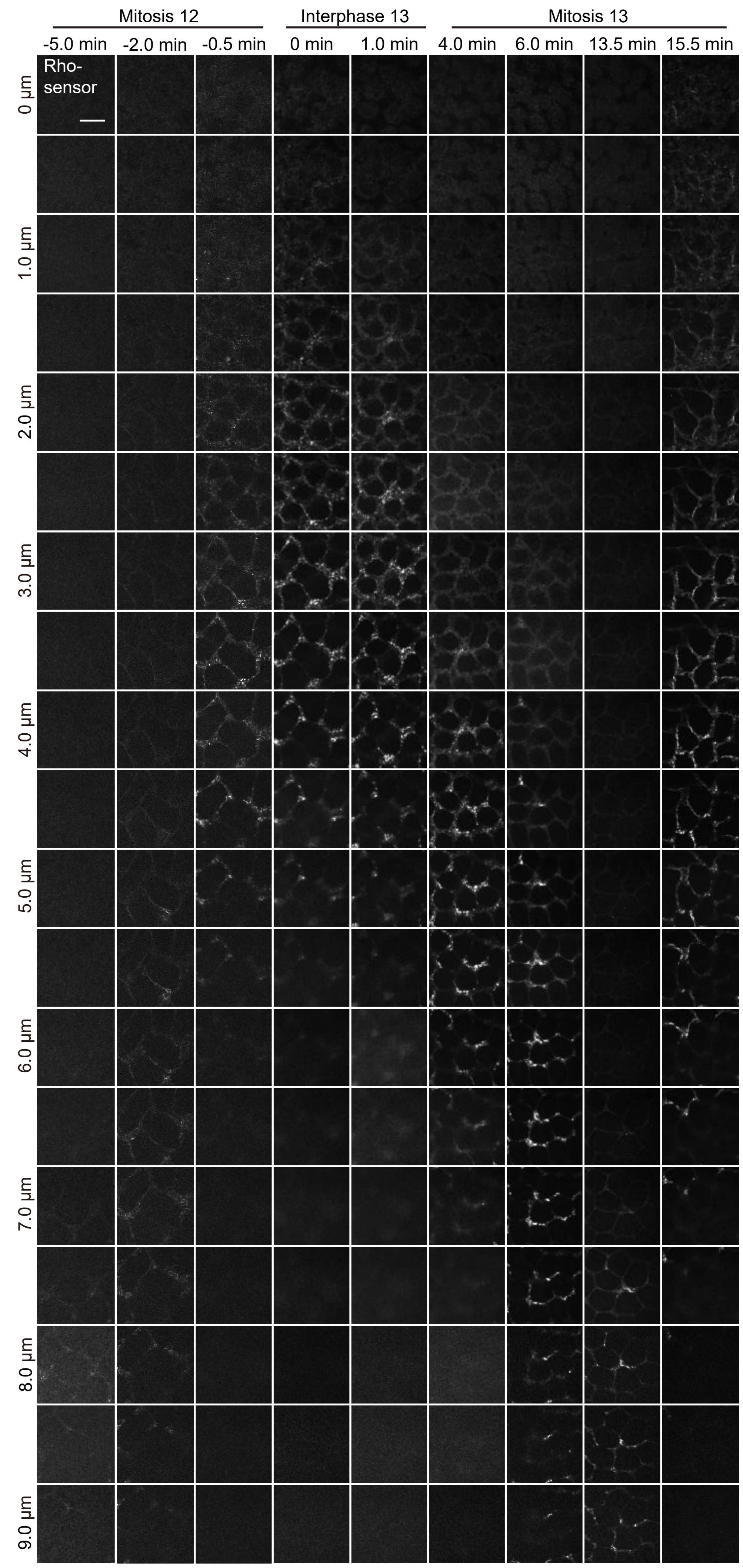

Fig. S1. Spatialtemporal localization of Rho sensor during interphase and mitosis. Live imaging of a syncytial blastoderm embryo expressing the Rho sensor tagged with GFP. Axial position and time are as indicated. Time 0 was defined by the localization of the Rho sensor to new furrows. Scale bar 10 µm.

Journal of Cell Science • Supplementary information

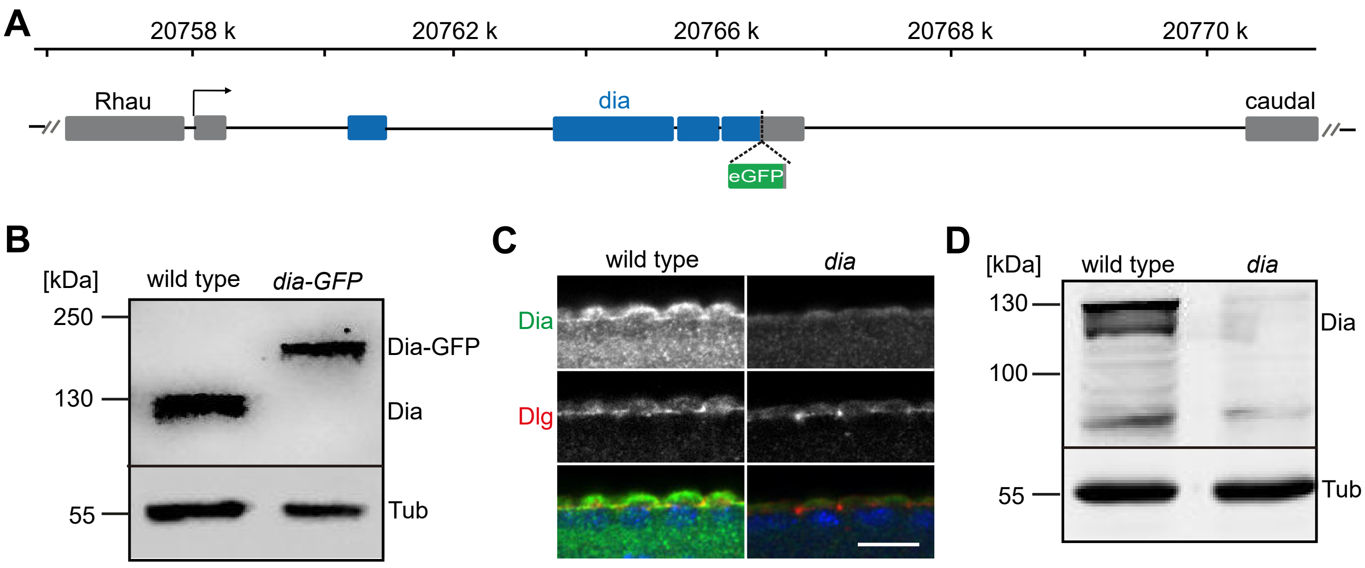

Fig. S2. GFP tagging of Dia. (A) Scheme of the genetic region of dia with eGFP insertion. **(B)** Western blot with wild type and Dia-GFP embryonic lysates stained for Dia (upper blot) and Tubulin as loading control (lower blot). **(C)** Wild type and dia embryos in interphase 13 fixed and stained for Dia (grey/green), Dlg (grey/red) and DNA (blue). Sagittal sections **(D)** Western blot with wild type and *dia* embryonic lysates stained for Dia (upper blot) and Tubulin as loading control (lower blot). Scale bar 10 µm.

Journal of Cell Science • Supplementary information

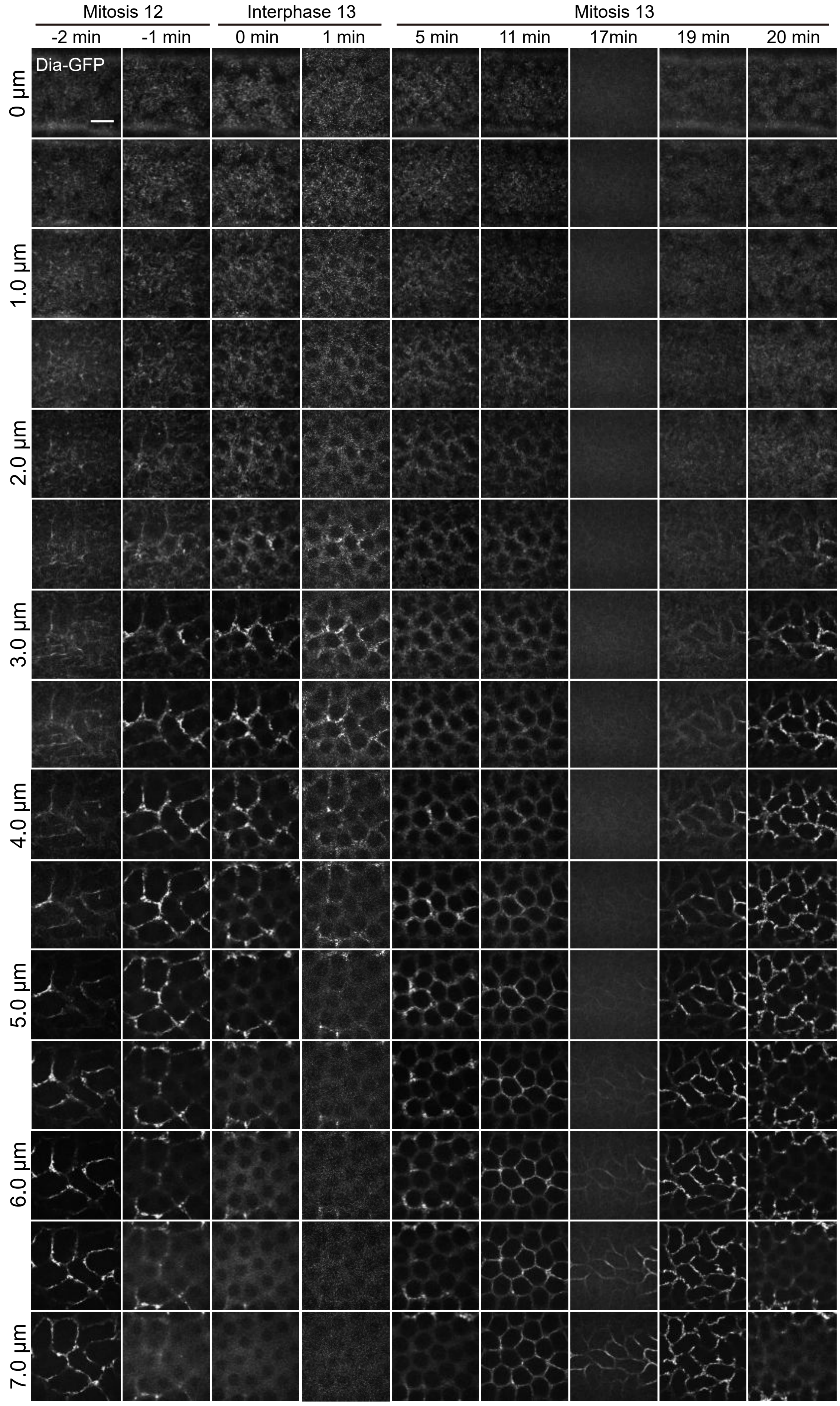

Fig. S3. Dia-GFP is enriched in intercaps and basal tips of metaphase furrow. Images from a movie of a syncytial blastoderm embryo expressing Dia-GFP. Axial position and time are as indicated. Time 0 was defined by the localization of Dia-GFP to new furrows. Scale bar 10 µm.

Journal of Cell Science • Supplementary information

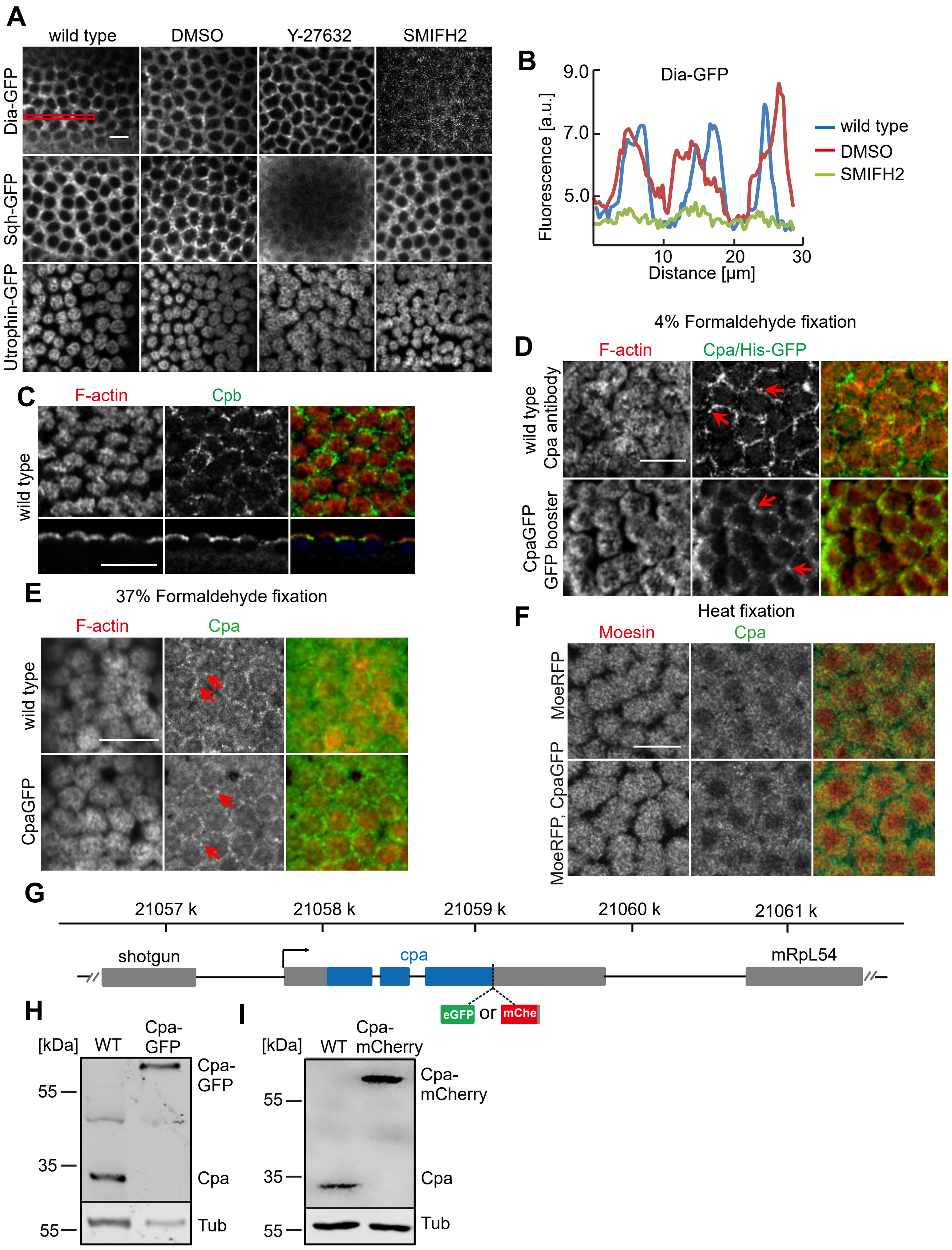

Fig. S4. Injection of inhibitors diminishes fluorescence of Dia-GFP and Sqh-GFP. (A) Embryos expressing indica-ted tagged proteins were injected with the indicated drugs. Images from live embryos during interphase 13. **(B)** Normali-zed Dia-GFP fluorescence intensity at the intercap domain. **(C)** Wild type embryo during interphase 13 fixed and stained for F-actin (grey/red), Cpb (grey/green) and DNA (blue). Upper panels shows frontal sections, lower panel sagittal views. **(D, E)** Histone2Av-GFP and CpaGFP embryos fixed with 4% **(D)** or 37% **(E)** formaldehyde and stained for Cpa antibody or GFP antibody as indicated. **(F)** Embryos expressing indicated tagged proteins were heat-fixed and stained for RFP and Cpa. Arrows in red indicate Cpa punctae. **(G)** Cpa locus on the second chromosome with eGFP or mCherry insertions as indicated. **(H, I)** Western blots with wild type and CpaGFP or Cpa-mCherry embryonic lysates probed for Cpa (upper blot) and α-Tubulin as loading control. Scale bars 10 µm.

Journal of Cell Science • Supplementary information

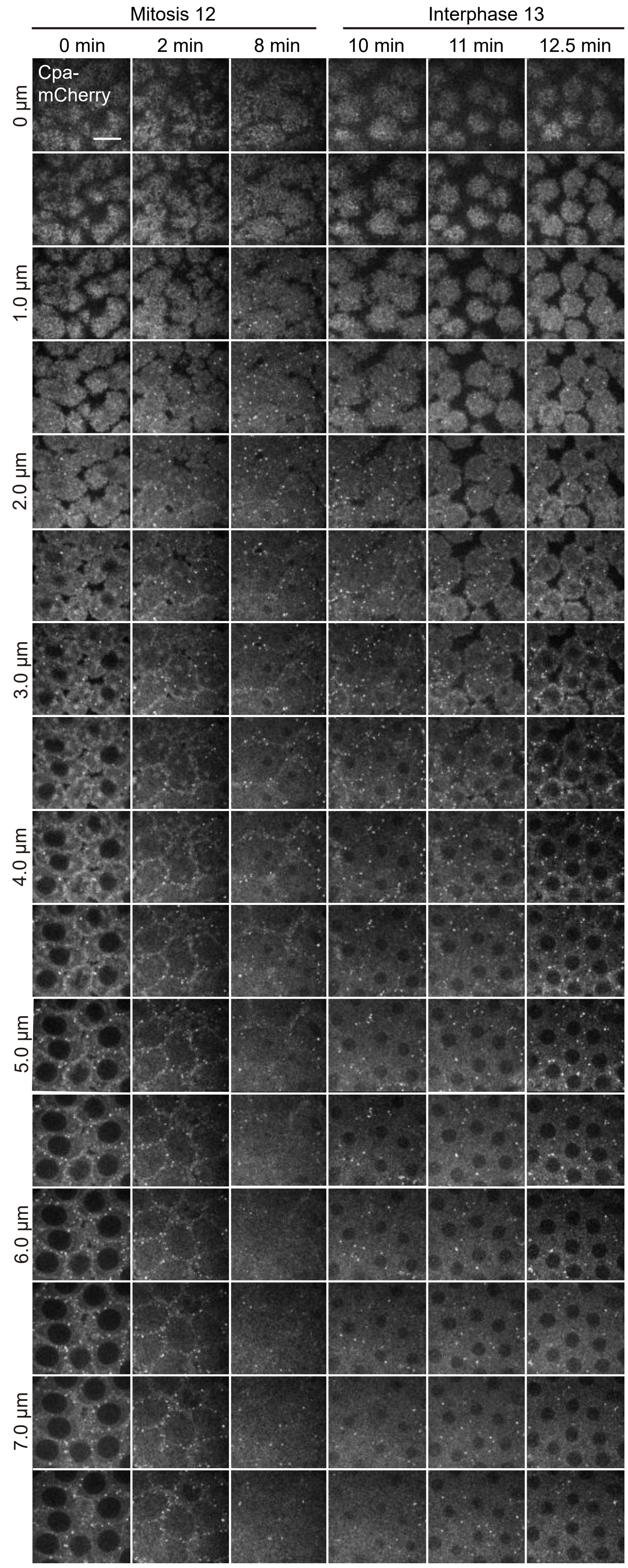

Fig. S5. Dynamics of Cpa-mCherry during mitosis and interphase. Live syncytial blastoderm embryos expressing Cpa-mCherry from mitosis 12 to interphase 13. Axial position and time as indicated. Time 0 was defined by the expansion of the cap region at the begin-ning of mitosis. Scale bar 10 µm.

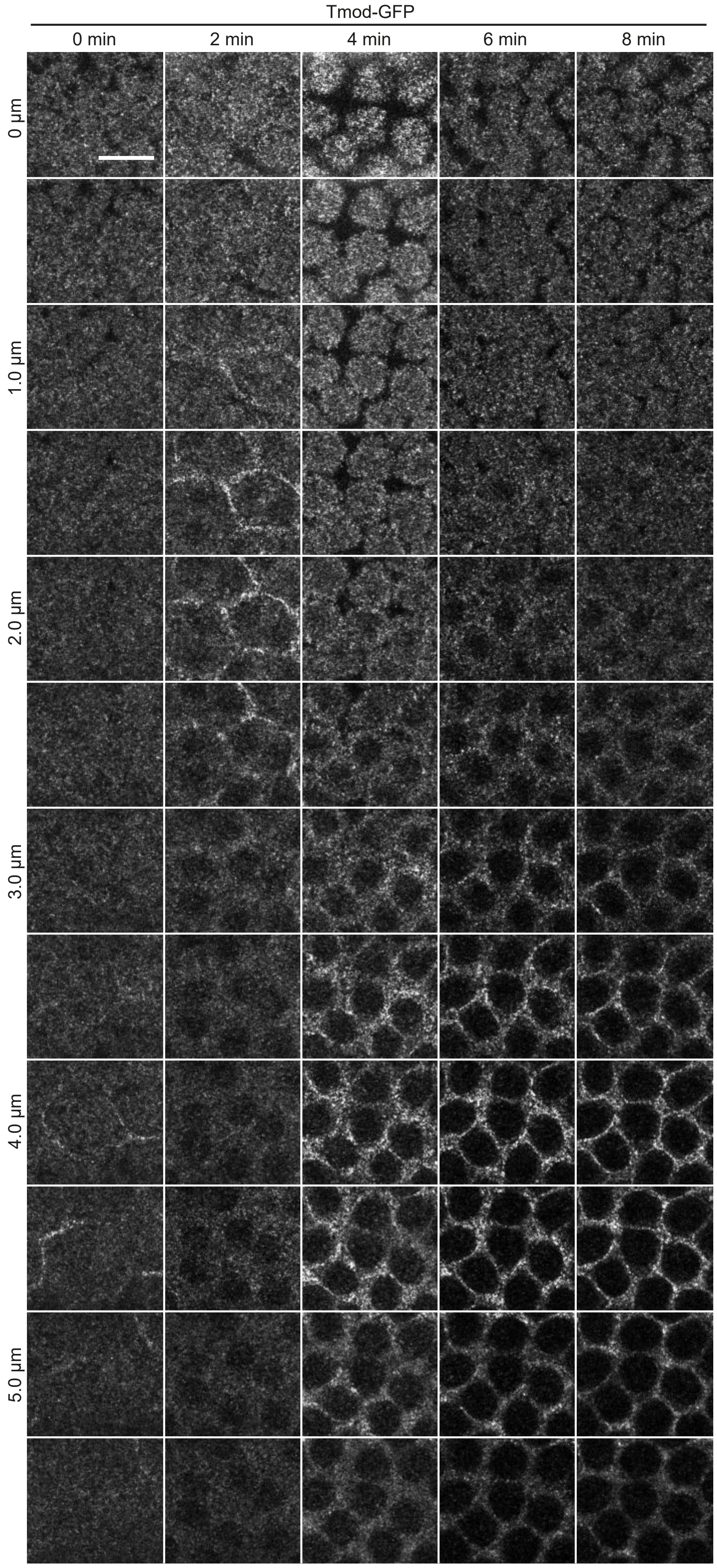

Fig. S6. Dynamics of Tmod-GFP during mitosis and interphase. Live syncytial blasto-derm embryos expressing Tmod-GFP from mitosis 12 to inter-phase 13. Axial position and time as indicated. Time 0 was defined by the expansion of the cap region at the beginning of mitosis. Scale bar 10 µm.

Table S1. Fly stocks

**Fly stocks**	**Reference/Source**
Ced-12/ELMO^{c06760}	Drosophila stock center, Bloomington # 17781
dia^{sy5}	Yan et al., 2013
Cpa-GFP	This work
Cpa-mCherry	This work
Dia-GFP	This work
Histone2Av-GFP	Clarkson and Saint, 1999
Moesin-RFP	Großhans lab
MyoII 3xGFP	Pinheiro et al., 2017
Rho sensor	Munjal et al., 2015
Tmod-GFP	Drosophila stock center, Bloomington # 59303
Utrophin-GFP	Rauzi et al., 2010

Table S2. Antibodies, stains and inhibitors

**Antibody**	**Dilution**	**Reference/Source**
mouse anti-α-Tubulin	1:50000 (WB)	B512, Sigma
rabbit anti-Cpa	1:200 (IHC)<br>1:2000 (WB)	Amândio et al., 2014, this work
rabbit anti-Cpb	1:200	Amândio et al., 2014
Rabbit anti-Dia	1:1000 (IHC)	Großhans et al., 2005
guinea pig anti-Dia	1:5000 (WB)	Großhans et al., 2005
Phalloidin-Alexa647	1:1000	Thermo Fisher (# A12379)
GFP-booster-Atto488	1:500 (IHC)	Chromotek, (# gba488)
DAPI	1:250 (IHC), 0.2 µg/ml	Thermo Fisher (# D1306)
SMIFH2	2 mM in DMSO	Abcam (# ab218296)
Y-27632	10 mM in water	Sigma (# Y0503)

Journal of Cell Science • Supplementary information
