# Supplementary material for: Dia- and Rok-dependent enrichment of capping proteins in a cortical region
Source: J Cell Sci. 2021 Nov 5;134(21):jcs258973. doi: 10.1242/jcs.258973 (PMC8627554; doi:10.1242/jcs.258973)
Supplement: Reviewer comments [file joces-134-258973_review_history.pdf]

Dia- and Rok-dependent enrichment of capping proteins in a cortical region

Anja Schmidt, Long Li, Zhiyi Lv, Shuling Yan and Jöerg Grosshans
DOI: 10.1242/jcs.258973

Editor: Michael Way

Review timeline

Original submission:	29 May 2021
Editorial decision:	29 June 2021
First revision received:	19 August 2021
Editorial decision:	20 September 2021
Second revision received:	23 September 2021
Accepted:	24 September 2021

Original submission

First decision letter

MS ID#: JOCES/2021/258973

MS TITLE: Dia- and MyoII-dependent enrichment of capping proteins in cortical intercap region

AUTHORS: Anja Schmidt, Long Li, Zhiyi Lv, Shuling Yan, and Jöerg Grosshans
ARTICLE TYPE: Research Article

We have now reached a decision on the above manuscript.

To see the reviewers' reports and a copy of this decision letter, please go to: https://submit-jcs.biologists.organd click on the 'Manuscripts with Decisions' queue in the Author Area. (Corresponding author only has access to reviews.)

As you will see, although the quality of your study was appreciated, it still received very mixed reviews. In particular, reviewer 2 feels the study is too preliminary and does recommend revision because it does not represent an advance over previous work. In contrast, the other two reviewers are mildly supportive but do raise a number of criticisms. I think they have raised some valid points that will need to be addressed including a much better description and quantification of puncta variability and what it might mean. In revising your study you should also try to highlight and discuss the new insights from your study to address reviewer 2's concerns. If you think that you can deal satisfactorily with these issue, I would be pleased to see a revised manuscript. We would then return it to the reviewers.

We are aware that you may be experiencing disruption to the normal running of your lab that makes experimental revisions challenging. If it would be helpful, we encourage you to contact us to discuss your revision in greater detail. Please send us a point-by-point response indicating where you are able to address concerns raised (either experimentally or by changes to the text) and where you will not be able to do so within the normal timeframe of a revision. We will then provide further guidance. Please also note that we are happy to extend revision timeframes as necessary.

© 2021. Published by The Company of Biologists under the terms of the Creative Commons Attribution License (https://creativecommons.org/licenses/by/4.0/).

Please ensure that you clearly highlight all changes made in the revised manuscript. Please avoid using 'Tracked changes' in Word files as these are lost in PDF conversion.

I should be grateful if you would also provide a point-by-point response detailing how you have dealt with the points raised by the reviewers in the 'Response to Reviewers' box. Please attend to all of the reviewers' comments. If you do not agree with any of their criticisms or suggestions please explain clearly why this is so.

Reviewer 1

Advance summary and potential significance to field

In this manuscript, Schmidt et al. examine the impact of the formin Dia and motor protein MyoII on Cpa distribution in the early Drosophila embryo. The authors show that along with Rho1 signaling, Cpa and Cpb are enriched at the intercaps where Dia and MyoII were previously reported to be enriched (Afshar et al., 2000; Royou et al., 2002). In addition to this, the authors detail the distribution of Cpa in the syncytial blastoderm, which has not been described before. They go on to show that this Cpa enrichment is dependent on Dia and dRok activity and is likely a result of roles of Dia and MyoII in actin organization. Additionally, the authors identify interesting Cpa-containing clusters that with more investigation could provide further insight into actin organization and regulation in the syncytial embryo.

Comments for the author

Overall, the manuscript is well-written and straightforward with the results and conclusions. Moreover, the authors generated fly lines with fluorescently tagged Cpa and Dia among other reagents that will be useful contributions to the community.

• Using the Rho kinase inhibitor Y-27632 can affect cellular processes beyond inhibiting MyoII activity.
ROCK inhibition has been shown to suppress cofilin phosphorylation through LimK in mammalian cells (Thirone et al. 2009), which could have an effect on actin organization that is independent of myosin. If possible, the authors should consider using a MyoII mutant or MyoII RNAi in conjunction with the drug inhibition to make the data more convincing. Also, the figure citations on line 166 and 179 are incorrect- it should be Suppl. Figure S4 not Suppl. Figure S6?

• It would be helpful to have a more detailed methodology for the line profile measurements on actin caps (i.e. how were the decisions made of where to draw the line? Is it from the border of the cap to the nucleus?)
In Figure 3A, it looks like the line was drawn from the border to the nucleus in WT but from the nucleus to another nucleus in the dia mutant.

• Enrichment of capping protein could mean an enrichment of F-actin plus ends or a greater number of short filaments (plus and minus ends). A F-actin minus-end marker like Tropomodulin could help provide insight into the possibilities the authors raise in the discussion- whether the clusters are actin organized into a certain formation similar to what has been seen previously in vitro, or if the clusters are an enrichment of plus-ends, generated by Dia.

Minor/typos:
• Line 95: ‘worthnoting’ should have a space.
• Line 107: Should this be 'dia dependent manner' and not 'dia depending manner'?

Reviewer 2

Advance summary and potential significance to field

In this study Schmidt and coworkers report on the localisation of actin capping proteins alpha during early Drosophila syncytial stages. They found that the actin capping protein alpha localizes in a polarizes manner to invaginating furrow regions where the formin Diaphanous (Dia) and the molecular motor MyosinII are also enriched, while it is depleted form cortical apical regions. Using

© 2021. Published by The Company of Biologists under the terms of the Creative Commons Attribution License (https://creativecommons.org/licenses/by/4.0/).

a combination of drug and genetic perturbations they demonstrate that this localisation depends on both Dia and Myosin activity. It was previously known that Dia, which binds preferentially to plus ends, localises to invaginating furrows.
Therefore, it is not particularly surprising that CAP alpha e beta are enriched in this region. Although this study is well conducted (with endogenous tagging of both Dia and CAP α I am not sure at this stage the message delivered is sufficiently elaborated to justify publication. I am failing to see either description of a novel interesting biological process or functional dissection of an established molecular process that would make this study ready for publication.

Comments for the author

I think this study is too preliminary to provide detailed suggestions on how to move forward towards a revised version.

Reviewer 3

Advance summary and potential significance to field

The early Drosophila embryo is a syncytial cell, in which the nuclei at the periphery divide several times. Nuclei are spatially organized in distinct cortical “caps” by a meshwork of actin, which are separated from each other by “intercaps”.
In this manuscript the authors show by using a genetically encoded Rho sensor that the intercaps are also enriched in increased Rho-signaling and, besides MyoII, in another Rho target, Dia. They further demonstrate an accumulation of Capping protein (Cpa) alpha and beta, markers of actin plus-ends, at the intercaps. This was unexpected, since the intercaps contain very little actin. Enrichment of Cpa is dependent on Dia and MyoII activity. This led the authors to suggest a model in which Rho-signaling controls Cpa accumulation and hence the organization of the cortical actin in the intercaps downstream of Dia and MyoII. Whether Cpa enrichment is linked to plus ends of actin in intercaps is discussed, but remains to be explored.

Comments for the author

Overall, this is a nice piece of work, which includes in-vivo analysis and extends the knowledge on the regulation of a process that ensures the proper formation of the Drosophila blastoderm. However, the conclusion drawn from some of the data cannot always be followed, in particular when it comes to the localization of proteins (see below, no. 8). The discussion gives the impression that this pathway is very specific to this particular process during Drosophila embryogenesis.

Below I summarize a few comments on the manuscript:
1. The discussion falls somewhat short to tell the reader whether the observations are very specific to the early embryo or whether this pathway may also work in other systems.

2. In several of the figures (e.g. Fig. 1), it would have been helpful to have a nuclear staining as well. This may be important, since particular in several horizonal sections it is not clear at which level the image has been taken (e.g. Fig. 5E)

3. Line 100-106 and Fig 1: It is not clear whether the Rho-sensor, Dia-GFP and MyoII co-localize at both stages.

4. Line 137-139: The meaning of this sentence remains somewhat obscure. On the one hand I have got the impression that the Dia-GFP and Cpa-Cherry punctae reflect the real localization of the respective proteins. But, as stated in this sentence, they are distinct, i.e. they do not co-localize.

5. Line 158: Is the “prominent accumulation of Cpa-GFP punctae” completely lost or are the punctae now somewhere else?

6. Line 187: “We did not detect a difference in staining with Phalloidin”. Where did they measure, in caps? in intercaps?

© 2021. Published by The Company of Biologists under the terms of the Creative Commons Attribution License (https://creativecommons.org/licenses/by/4.0/).

7. Line 196, Fig. 5D: My impression is that in dia mutant embryos the variability is much higher.

8. Line 211-213 and Fig. 5E: I think they need to better define “punctae”. There are big ones and small ones, as well as “diffuse weak staining” (line 138), but the text here remains vague concerning the difference between “diffuse weak staining” and “less prominent punctae”.

Minor points:
- Both the title and the summary can only be fully understood by people working in this field, who know what caps and intercaps are.
- Line 186: “we quantitatived”. I guess they mean quantified?

First revision

Author response to reviewers' comments

Point-by-point response to the reviewer’s comments

Reviewer 1
•Using the Rho kinase inhibitor Y-27632 can affect cellular processes beyond inhibiting MyoII activity. ROCK inhibition has been shown to suppress cofilin phosphorylation through LimK in mammalian cells (Thirone et al. 2009), which could have an effect on actin organization that is independent of myosin. If possible, the authors should consider using a MyoII mutant or MyoII RNAi in conjunction with the drug inhibition to make the data more convincing. Also, the figure citations on line 166 and 179 are incorrect- it should be Suppl. Figure S4 not Suppl. Figure S6?
We corrected our statements and conclusions by naming “Rok” or “Rok/MyoII” instead of MyoII. There is no doubt, that Rok potentially phosphorylates proteins in addition to MyoII regulatory light chain. And inhibition of Rok is certainly not the same as a specific loss of MyoII activity, although it often comes very close depending on the experimental system. We also explicitly stated that other Rok targets may contribute to the observed phenotypes: “We cannot exclude that other targets of Rok beside MyoII contribute to the observed phenotypes.”
We chose not to use RNAi to assess the function of MyoII directly. Such an experiment is difficult in syncytial embryos, because preceding stages in oogenesis and preblastoderm embryos will also be affected.
We checked and corrected figure citations.

•It would be helpful to have a more detailed methodology for the line profile measurements on actin caps (i.e. how were the decisions made of where to draw the line? Is it from the border of the cap to the nucleus?) In Figure 3A, it looks like the line was drawn from the border to the nucleus in WT but from the nucleus to another nucleus in the dia mutant.
We described the methods in more detail in the respective results part.

•Enrichment of capping protein could mean an enrichment of F-actin plus ends or a greater number of short filaments (plus and minus ends). A F-actin minus-end marker like Tropomodulin could help provide insight into the possibilities the authors raise in the discussion- whether the clusters are actin organized into a certain formation similar to what has been seen previously in vitro, or if the clusters are an enrichment of plus-ends, generated by Dia.
At an initial stage of the study several years ago, we imaged embryos expressing the minus end marker Tropomodulin-GFP. At that time we did not detect a signal in syncytial embryos. Following the suggestion of the reviewer, we have now repeated this experiment with a more sensitive microscope (LSM980), which has been available to us since early this year. We detected an overall cortical signal and an enrichment in the intercap region comparable to Cpa in fixed specimen. We did not observe punctae, which are so prominent in living Cpa-GFP embryos. We included images (Figure 2F, Suppl Figure S6) from living embryos expressing Tmod-GFP but did not test the dependance on dia or Rok/MyoII.

Minor/typos:

© 2021. Published by The Company of Biologists under the terms of the Creative Commons Attribution License (https://creativecommons.org/licenses/by/4.0/).

•Line 95: ‘worthnoting’ should have a space.
•Line 107: Should this be 'dia dependent manner' and not 'dia depending manner'?
We corrected the typos

Reviewer 3

Overall, this is a nice piece of work, which includes in-vivo analysis and extends the knowledge on the regulation of a process that ensures the proper formation of the Drosophila blastoderm. However, the conclusion drawn from some of the data cannot always be followed, in particular when it comes to the localization of proteins (see below, no. 8).
We will recheck our conclusions and adjust where appropriate.
The discussion gives the impression that this pathway is very specific to this particular process during Drosophila embryogenesis.
We added a paragraph at the beginning of the discussion, where we speculate that a restricted distribution of Cpa may also be observed in other cell type and species, given the evolutionary conservation of the proteins involved.

Below I summarize a few comments on the manuscript:
1.The discussion falls somewhat short to tell the reader whether the observations are very specific to the early embryo or whether this pathway may also work in other systems.
We hesitate to generalize too much, since we conducted the experiments only with syncytial embryos and it remains to be analyzed whether our findings hold true at other stages and experimental systems. We added a paragraph at the beginning of the discussion, where we speculate that a restricted distribution of Cpa may also be observed in other cell type and species, given the evolutionary conservation of the proteins involved.

2.In several of the figures (e.g. Fig. 1), it would have been helpful to have a nuclear staining as well. This may be important, since particular in several horizonal sections it is not clear at which level the image has been taken (e.g. Fig. 5E)
We added the channel with DAPI staining to the images of fixed specimen (Figure 6E) or marked the position of the nuclei by dashed lines. We do not have images with nuclear staining in live imaging. We marked the nuclei as recognizable by cytoplasmic exclusion in images from live imaging where possible (Fig. 1D, 4D).

3.Line 100-106 and Fig 1: It is not clear whether the Rho-sensor, Dia-GFP and MyoII co-localize at both stages.
This is an interesting issue, which we will certainly address in the future. We have not yet conducted double labeling of Rho sensor, Dia-GFP and MyoII-GFP in live embryos. From the morphology it is clear that all three labels are within the intercap region during interphase and at the basal tip of the metaphase furrow during mitosis. It remains unclear whether the particulate pattern overlaps in the submicrometer scale. For that, super resolution microscopy should be employed.

4.Line 137-139: The meaning of this sentence remains somewhat obscure. On the one hand I have got the impression that the Dia-GFP and Cpa-Cherry punctae reflect the real localization of the respective proteins. But, as stated in this sentence, they are distinct, i.e. they do not co-localize.
We rephrased this issue.

5.Line 158: Is the “prominent accumulation of Cpa-GFP punctae” completely lost or are the punctae now somewhere else?
We included a new figure 4 with live imaging and Cpa-GFP dynamics. We quantified the density of Cpa-GFP punctae along the apical-basal axis in wild type and *dia* embryos. We find that the Cpa-GFP punctae accumulate at a basal position within the first few minutes of interphase, whereas they remain widely spread in dia mutants.

6.Line 187: “We did not detect a difference in staining with Phalloidin”. Where did they measure, in caps? in intercaps?
We clarified this in the results part.

7.Line 196, Fig. 5D: My impression is that in dia mutant embryos the variability is much higher.

© 2021. Published by The Company of Biologists under the terms of the Creative Commons Attribution License (https://creativecommons.org/licenses/by/4.0/).

This is in fact the case. We mentioned this in the text.
L243 "However, we could detect a higher variability in syncytial blastoderm *dia* mutants compared to the fluorescence recovery in wild type embryos."

8.Line 211-213 and Fig. 5E: I think they need to better define "punctae". There are big ones and small ones, as well as "diffuse weak staining" (line 138), but the text here remains vague concerning the difference between "diffuse weak staining" and "less prominent punctae".
We are more stringent with our description.
We introduce the punctae as being variable in intensity.
L126 "we detected prominent punctae of a wide range of intensities,"

Minor points:
•Both the title and the summary can only be fully understood by people working in this field, who know what caps and intercaps are.
We revised the title by substituting MyoII with Rok and intercaps with a cortical region.
New title: "Dia- and Rok-dependent enrichment of capping proteins in a cortical region"

•Line 186: "we quantitatived". I guess they mean quantified?
We corrected this typo.

•

Second decision letter

MS ID#: JOCES/2021/258973

MS TITLE: Dia- and Rok-dependent enrichment of capping proteins in a cortical region

AUTHORS: Anja Schmidt, Long Li, Zhiyi Lv, Shuling Yan, and Jöerg Grosshans
ARTICLE TYPE: Research Article

We have now reached a decision on the above manuscript.

To see the reviewers' reports and a copy of this decision letter, please go to: https://submit-jcs.biologists.organd click on the 'Manuscripts with Decisions' queue in the Author Area. (Corresponding author only has access to reviews.)

As you will see, the reviewers gave favourable reports but raised some minor points that will require text amendments to your manuscript. I hope that you will be able to carry these out because I would like to be able to accept your paper. Basically, it is easier to do these minor corrections now rather than at the proof stage..

We are aware that you may be experiencing disruption to the normal running of your lab that makes experimental revisions challenging. If it would be helpful, we encourage you to contact us to discuss your revision in greater detail. Please send us a point-by-point response indicating where you are able to address concerns raised (either experimentally or by changes to the text) and where you will not be able to do so within the normal timeframe of a revision. We will then provide further guidance. Please also note that we are happy to extend revision timeframes as necessary.

Please ensure that you clearly highlight all changes made in the revised manuscript. Please avoid using 'Tracked changes' in Word files as these are lost in PDF conversion.

I should be grateful if you would also provide a point-by-point response detailing how you have dealt with the points raised by the reviewers in the 'Response to Reviewers' box. Please attend to all of the reviewers' comments. If you do not agree with any of their criticisms or suggestions please explain clearly why this is so.

© 2021. Published by The Company of Biologists under the terms of the Creative Commons Attribution License (https://creativecommons.org/licenses/by/4.0/).

Reviewer 1

Advance summary and potential significance to field

The authors show that along with Rho1 signaling, Cpa and Cpb are enriched at the intercaps where Dia and MyoII were previously reported to be enriched (Afshar et al., 2000; Royou et al., 2002). The authors carefully describe the distribution of capping protein (Cpa) in the syncytial blastoderm, which has not been done. They show that this Cpa enrichment is dependent on Dia and dRok activity.

Comments for the author

Main comment: The authors have included new data into the manuscript (e.g. Tropomodulin localization), but did not integrate this result into the Discussion. It appears that there is an enrichment of both barbed and pointed ends in the intercap region. I was looking for the authors to provide an interpretation (speculation) for what this could represent? What came to my mind is that contractility could fragment F-actin, which could generate both an enrichment of both ends (Murrell et al., PNAS, 2012).

Minor:
1) Clarity of the first sentence of the summary can be improved. Recommend splitting into multiple sentences.
2) General: Because there are multiple Rho GTPases, recommend being specific and saying RhoA or Rho1 signaling.
3) Line 98: need space in worthnoting (could be PDF conversion error).
4) Line 112: ‘dia depending’ -> dia dependent.
5) Typo in line 174 (broadly should be broad).
6) Typo in line 254 (dispaly should be display).
7) Formatting in line 95 (CrispR to CRISPR?)
8) Line 604: missing p in front of inequality.

Reviewer 3

Advance summary and potential significance to field

In this manuscript the authors show by using a genetically encoded Rho sensor that the intercaps are also enriched in increased Rho-signaling and, besides MyoII, in another Rho target, Dia. They further demonstrate an accumulation of Capping protein (Cpa) alpha and beta, markers of actin plus-ends, at the intercaps. This was unexpected, since the intercaps contain very little actin. Enrichment of Cpa is dependent on Dia and MyoII activity. This led the authors to suggest a model in which Rho-signaling controls Cpa accumulation and hence the organization of the cortical actin in the intercaps downstream of Dia and MyoII. Overall, this is a nice piece of work, which includes in-vivo analysis and extends the knowledge on the regulation of a process that ensures the proper formation of the Drosophila blastoderm.

Comments for the author

The authors have carefully addressed all points raised in my previous review, satisfactorily replied to them, and modified where required.

Second revision

Author response to reviewers' comments

Reviewer 1 Comments for the author

© 2021. Published by The Company of Biologists under the terms of the Creative Commons Attribution License (https://creativecommons.org/licenses/by/4.0/).

Main comment: The authors have included new data into the manuscript (e.g. Tropomodulin localization), but did not integrate this result into the Discussion. It appears that there is an enrichment of both barbed and pointed ends in the intercap region. I was looking for the authors to provide an interpretation (speculation) for what this could represent? What came to my mind is that contractility could fragment F-actin, which could generate both an enrichment of both ends (Murrell et al., PNAS, 2012).

We introduced the following part into the discussion:
Taking a simple-minded view with Cpa and Tmod as markers of filament ends, the observed enrichment can be interpreted as a preferential accumulation of F-actin plus and minus ends in the intercap region. Our stainings and measurements indicate that the ratio of filament ends versus total amount of F-actin is strongly increased in the intercap region. However, the interpretation and underlying causes may be more differentiated and complicated. One explanation is that filament ends are not equally accessible to the Cpa/Cpb complex and Tmod. For example, the cap region largely contains Arp2/3 dependent branched F-actin, which by structure contain fewer minus ends. The plus ends within the caps may have a reduced preference for Cpa binding or may be subject to competition from other, branched actin specific regulators. Alternatively, the filaments may be much shorter in the intercap than cap region. In vitro studies showed that Myosin-based contractility may lead to severing of actin filaments (Murrell and Gardel, 2012). A consequence of the Rho1 signaling and MyoII activation in the intercap region may be increased contractility and filament severing. Shorter filaments may also arise from dedicated severing proteins like cofilin or from increased actin turnover. Despite these complications, the observed enrichment of markers for filament ends indicates a specific feature of cortical actin organization at the intercap region involving plus ends.

Minor:
1)Clarity of the first sentence of the summary can be improved. Recommend splitting into multiple sentences.
Clarified: Rho signaling with its major targets the formin Dia, Rho kinase (Rok) and non-muscle myosin II control turnover, amount and contractility of actomyosin. Much less investigated has been a potential function for the distribution of F-actin plus and minus ends.....
2)General: Because there are multiple Rho GTPases, recommend being specific and saying RhoA or Rho1 signaling.
Changed to Rho1 / Rho1 sensor / Rho1 signaling when specifically referred to Drosophila
3)Line 98: need space in worthnoting (could be PDF conversion error).
corrected
4)Line 112: ‘dia depending’ -> dia dependent.
corrected
5)Typo in line 174 (broadly should be broad).
corrected
6)Typo in line 254 (dispaly should be display).
corrected
7)Formatting in line 95 (CrispR to CRISPR?)
corrected
8)Line 604: missing p in front of inequality.
corrected

Third decision letter

MS ID#: JOCES/2021/258973

MS TITLE: Dia- and Rok-dependent enrichment of capping proteins in a cortical region

AUTHORS: Anja Schmidt, Long Li, Zhiyi Lv, Shuling Yan, and Jöerg Grosshans
ARTICLE TYPE: Research Article

© 2021. Published by The Company of Biologists under the terms of the Creative Commons Attribution License (https://creativecommons.org/licenses/by/4.0/).

I am happy to tell you that your manuscript has been accepted for publication in Journal of Cell Science, pending standard ethics checks.

© 2021. Published by The Company of Biologists under the terms of the Creative Commons Attribution License (https://creativecommons.org/licenses/by/4.0/).
